# Supermolecular Structure of Poly(Butylene Terephthalate) Fibers Formed with the Addition of Reduced Graphene Oxide

**DOI:** 10.3390/polym12071456

**Published:** 2020-06-29

**Authors:** Czesław Ślusarczyk, Marta Sieradzka, Janusz Fabia, Ryszard Fryczkowski

**Affiliations:** Institute of Textile Engineering and Polymer Materials, University of Bielsko-Biala, Willowa 2, 43-309 Bielsko-Biala, Poland; msieradzka@ath.bielsko.pl (M.S.); jfabia@ath.bielsko.pl (J.F.); rfryczkowski@ath.bielsko.pl (R.F.)

**Keywords:** poly(butylene terephthalate), reduced graphene oxide, WAXS, SAXS, DSC, lamellar structure, crystalline structure, smectic mesophase

## Abstract

Nanocomposite fibers based on poly(butylene terephthalate) (PBT) and reduced graphene oxide (rGO) were prepared using a method able to disperse graphene in one step into a polymer matrix. The studies were performed for fibers containing four different concentrations of rGO at different take-up velocities. The supermolecular structures of the fibers at the crystallographic and lamellar levels were examined by means of calorimetric and X-ray scattering methods (DSC, WAXS, and SAXS). It was found that the fiber structure is mainly influenced by the take-up velocity. Fibers spun at low and medium take-up velocities contained a crystalline α-form, whereas the fibers spun at a high take-up velocity contained a smectic mesophase. During annealing, the smectic phase transformed into its α-form. The degree of transformation depended on the rGO content. Reduced graphene mainly hindered the crystallization of PBT by introducing steric obstacles confining the ordering of the macromolecules of PBT.

## 1. Introduction

Poly(butylene terephthalate) (PBT) is a commercially important polymeric material with a wide range of applications in bulk, fibers, and films [1]. It belongs to a homologous series of aromatic polyesters, with poly(ethylene terephthalate) (PET) and poly(trimethylene terephthalate) (PTT) as the most important representatives. It possesses some remarkable properties, such as a good strength and modulus at elevated temperatures, good chemical resistance, high dielectric strength and excellent electrical properties, and high heat and flame resistance [2,3]. The melting temperature of PBT is lower than that of PET; hence, it has some processing advantages over its chemical relative. In addition, PBT has a lower glass transition temperature, a faster crystallization rate, and approximately the same achievable maximum crystallinity as PET. As it crystallizes more rapidly than PET, it tends to be preferred for industrial scale molding. Furthermore, as a fiber, PBT is much more elastic and has excellent resilience and recovery from small deformations. It dyes easily with disperse dyes at the boil, unlike PET, and resists photooxidative yellowing [4].

As a member of the polyester family, PBT is widely used for engineering thermoplastics [5] or as a component in blends [6,7], copolymers [8,9,10], and composites [11,12,13]. However, for high-performance applications, PBT needs to be enhanced through combination with other polymers and the use of reinforcements. The following substances have been used as reinforcements for PBT: calcium carbonate [14], carbon black [15], glass fibers [16], carbon fibers [17], montmorillonite [18], carbon nanotubes [19,20], and thermotropic liquid crystal polymer [21]. Since graphene was discovered, attempts have been made to use it as a filler in nanocomposites, as manifested in numerous publications about graphene-based polymer nanocomposites [22,23,24,25]. Poly(butylene terephthalate) is also among the various polymer matrices enriched with this nanocomponent [26,27,28]. In the literature, graphene is described as a unique material with excellent mechanical properties, high thermal and electrical conductivity, and a high specific surface area [29,30,31]. For this reason, it is considered a nanomaterial with a wide range of potential applications, ranging from supercapacitors, transistors, sensors, and electrodes in solar cells to conductive inks and flexible touch screens [32,33,34]. The outstanding properties of graphene compared to pure polymers are reflected in graphene-based polymer composites which show superior thermal, mechanical, electrical, gas barrier, and flame-retardant properties [35,36,37].

The most important factors in the preparation of polymer nanocomposites, along with the addition of graphene, are obtaining adequate adhesion in the polymer/graphene system and a homogeneous dispersion of graphene in the polymer matrix [38]. The lack of adhesion between the filler and the polymer matrix can cause the formation of nanoparticle aggregates during the nanocomposite preparation. Besides this, it can cause an early failure at the interface, and thus result in changes in the physical properties of the final composite [39]. An advantage of graphene, compared with other reinforcements, is that it allows for significant changes in the properties of composites at very low percolation thresholds due to its ultrahigh aspect ratio [38,40]. The method used to obtain these nanocomposites is also of great importance for their properties. In situ polymerization, solvent processing, melt blending, and layer by layer assembly are among the most commonly used methods for producing nanocomposites [33,38,40]. As noted by M. Li et al. [41], by using the method of mixing components into the melt, it was possible to obtain a nanocomposite with graphene that was very well dispersed in the polyester matrix, even when its content was 7 wt %. The electrical volume resistivity of the nanocomposites decreased dramatically from ~10^18^ Ωcm to ~10^6^ Ωcm with a nanoadditive content of 3 wt % to 5 wt %. In situ polymerization is another method of obtaining a PBT/graphene nanocomposite, which has been described by P. Fabbri et al. [42]. According to the authors, polymerization is the best way to obtain a nanocomposite with nanoadditives which are perfectly dispersed in the polymer matrix. As explained, the homogeneity of the components is achieved precisely during the polymerization reaction due to the intercalation of the polymer chains between the graphene layers. The authors also investigated the thermal properties of nanocomposites using differential scanning calorimetry (DSC) and thermogravimetric analysis (TGA). Their research shows that an increase in graphene content does not significantly affect the degree of crystallinity and the crystallization temperature of PBT but does cause a significant increase in thermal stability. 

Poly(butylene terephthalate) is a polymer crystallizing in a triclinic crystallographic system that has two crystallographic forms, α and β, as well as a smectic liquid crystalline structure. The α crystallographic form creates a much more stable structure in which chains containing four methylene groups occur in the gauche-trans-gauche (gtg) conformation. The α-form can change into the β-form with an all-trans (ttt) conformation when the polymer is subjected to stress [43]. Nevertheless, the β-form is unstable because, after the removal of the stress, the β-form returns to the α-form [44,45]. The smectic phase occurs when amorphous PBT is stretched at room temperature and transforms into the α-form upon heating [45].

The crystallization mechanism of PBT has been studied using many methods, most recently using a combination of synchrotron nanofocus X-ray scattering and fast scanning chip calorimetry (FSC) [46,47,48,49]. These studies, however, focused on quiescent crystallization under isothermal conditions. Nevertheless, polymer processing (such as extrusion, injection molding, fiber spinning, and film blowing) undoubtedly involves different types of flow fields (shear, extension, and mixed). For these reasons, an understanding of flow-induced crystallization is essential because it determines the formation of hierarchical structures and the final properties of semicrystalline polymer products.

S. Colonna et al. [26] prepared nanocomposites by the ring-opening polymerization of cyclic butylene terephthalate oligomers in the presence of reduced graphene oxide or highly reduced graphene oxide. The authors demonstrated the influence of rGO on the crystallization behavior of PBT, which was confirmed by nonisothermal DSC and isothermal DSC experiments. Based on self-nucleation experiments, they concluded that rGO nanofillers have a supernucleating effect on polymer crystallization; i.e., they are better nucleating agents than the polymer self-nuclei. As the authors report, the highly reduced graphene oxide possesses a higher nucleation efficiency than reduced graphene oxide as a result of the determinant role of the chemical and physical structure of the graphitic structure on the nucleation of the PBT crystals.

The nucleation and crystallization processes of the PBT matrix as influenced by the functionalized graphene oxide was described by P. Qian et al. [27]. As the authors report, the introduction the functionalized graphene oxide contributes to the higher crystallization peak (T_cp_) of nanocomposites compared to neat PBT. An increment for T_cp_ is seen as the increase from 0.1 to 3.0 wt % in the content of nanofillers, showing a good nucleation ability. Nevertheless, the maximum value was attained for 1.5 wt % nanofiller. According to the authors, due to the functionalization of graphene oxide, nanofiller plays a heterogeneous nucleation role and also promotes the crystallization behavior of the PBT matrix at low content levels. 

In the present article, we describe the process for obtaining PBT fibers enriched with reduced graphene oxide (rGO). The fibers were formed from the melt at different take-up velocities. The main goal of these investigations was to examine the influence of both spinning conditions and reduced graphene oxide on the morphology, supermolecular structure, and thermal properties of the obtained PBT/rGO nanocomposite fibers and the crystallization behavior of the polymer matrix. To characterize the microstructure development during the formation of the fibers, X-ray diffraction methods (WAXS and SAXS) and differential scanning calorimetry (DSC) were used. The changes in the crystallinity, crystallite size, and parameters of the lamellar structure were discussed based on the nucleation mechanism under the confinements introduced by the rGO particles.

## 2. Materials and Methods

### 2.1. Materials

Poly(butylene terephthalate) (PBT) (Celanex®, Celanese) was supplied by RESINEX Poland Sp. z o.o., Warsaw, Poland. The reagents employed during the preparation of graphene oxide, i.e., graphite powder <20 µm (Sigma-Aldrich, Poznan, Poland) and 98% H_2_SO_4_, KMnO_4_, 30% H_2_O_2_, and 35–38% HCl, were supplied by Chempur S.A. (Piekary Slaskie, Poland) and used as received, without further purification. Graphene oxide was thermally reduced without any additional reagents. The lateral size of rGO was, on average, 16.7 µm. Based on the WAXS analysis, the interlayer distance and average number of sheets were determined, which were 0.37 nm and 6, respectively. The elemental composition of the obtained reduced graphene oxide was determined by EDS analysis and was 81.4 at.% C, 18.6 at.% O [50]. The detailed characteristics of rGO can be found in the Supplementary Materials (Figure S1).

### 2.2. Graphene Oxide Preparation

The process of graphite oxidation proceeded according to the following procedure [50]: 30 g of graphite was added into a beaker with 750 cm^3^ sulfuric acid (VI). The mixture was stirred for 1 h at room temperature. Next, the beaker was placed in an ice bath to lower the temperature of the reaction mixture (below 10 °C). After cooling, 90 g of KMnO_4_ was batched into the beaker in portions. After adding the oxidation agent, the mixture remained in the ice-bath for 10 more minutes. The oxidation reaction continued for 2.5 h, not exceeding 40 °C. After oxidation, the samples were washed with distilled water (900 cm^3^), warm distilled water (60 °C, 600 cm^3^), and a 3% aqueous solution of H_2_O_2_. The obtained graphene oxide was purified by the ions from the reagents used. For this purpose, graphene oxide was rinsed ten times with distilled water and 10% aqueous solution of HCl. The obtained graphene oxide was thermally reduced in the next step.

### 2.3. Reduced Graphene Oxide Preparation

The prepared graphene oxide was thermally reduced by micro-explosion. For this purpose, partially dried GO was placed in a thermal reduction chamber that was blown with an inert gas (nitrogen). The graphene oxide in the chamber was heated at a rate of approximately 30 °C/min until the micro-explosion occurred. Thermal reduction was carried out until the process ceased to take place quickly. The final process temperature, which was measured after the completion of the micro-explosion, did not exceed 300 °C. Reduced graphene oxide was obtained as a fluffy, black powder with a bulk density of approximately 12 g/dm^3^ [50]. 

### 2.4. Preparation of PBT/rGO Fibers

PBT/rGO nanocomposites were obtained in a two-step process. The first stage involved the preparation of a masterbatch containing 10 wt % rGO. The polymer and nanoadditive were dried at 80 °C for 24 h before processing. The masterbatch was prepared using a Zamak Mercator EHP-2x16S co-rotating twin-screw extruder (Skawina, Poland) with a screw diameter of 15.8 mm and a length/diameter ratio (L/D) of 40. The temperature range for ten zones, from the feed zone to the head, ranged from 140 °C to 250 °C. Solidification of the extruded polymer stream took place as a result of heat exchange with the air. During the extrusion process, a filament was obtained, which was then granulated, and the masterbatch prepared in this way was used in the second stage of nanocomposite production. 

The fibers were formed using a Zamak Mercator extruder whose spinning nozzle contained 32 holes with a diameter of ~0.2 mm. The previously prepared masterbatch and pure polymer, which were also dried at 80 °C for 24 h, were used for the production of the fibers. The nanocomposite components were mixed in the proportions required to obtain the following contents of reduced graphene oxide in a final product of 0.5 wt %, 1 wt %, 1.5 wt %, and 2 wt %, which were marked as PBT + 0.5rGO, PBT + 1rGO, PBT + 1.5rGO, and PBT + 2rGO, respectively. Pure poly(butylene terephthalate) fibers (marked as PBT) were also prepared. 

The take-up velocity of the fibers was controlled by a Zamak Mercator RW1000 fiber stretching machine, and the individual speeds were 50 m/min, 100 m/min, 200 m/min, 400 m/min, 600 m/min, and 800 m/min, respectively. Gravity spun fibers, formed without the use of a stretching device, were also obtained. The take-up velocity is specified in the article as 0 m/min by convention. Each sample was marked with the take-up velocity at which it was formed—for example, PBT+1.5rGO-V600. This means that the sample containing 1.5 wt % of rGO was formed at a take-up velocity of 600 m/min.

### 2.5. Methods

The morphology of the samples was observed using a JEOL JSM 5500LV scanning electron microscope (SEM, JEOL Ltd., Tokyo, Japan). Before the images were obtained, all samples were coated with gold ~10 nm at 30 mA using a Leica EM ACE200 sputter coater (Wetzlar, Germany).

Differential scanning calorimetry (DSC) was performed using a Universal V4.5A TA Instruments device (New Castle, DE, USA)). The samples were heated from 0 to 260 °C with a 20 °C/min heating rate. Purging of nitrogen was performed at 40 ml/min.

The wide-angle X-ray scattering (WAXS) investigations were carried out with a URD-65 Seifert (Rich. Seifert & Co. Röntgenwertk, Ahrensburg, Germany) diffractometer. CuKα radiation was used at 40 kV and 30 mA. Monochromatization of the beam was obtained by means of a graphite crystal monochromator, placed in the diffracted beam path. A scintillation counter was used as a detector. Investigations were performed at angles from 3° to 60°, in steps of 0.1°. The investigated samples were powdered and pressed into a sample holder. The WAXS diffraction curves of the samples were deconvoluted into crystalline and amorphous scattering components using the profile fitting program WAXSFIT [51]. Each peak was modeled using a Gaussian–Cauchy peak shape. The crystallinity index was calculated as the ratio of the area under the crystalline peaks to the total area of the scattering curve. The lateral crystal sizes were deduced from the Scherrer equation, D_hkl_ = Kλ/(β_hkl_ cos θ), where the crystallite shape factor K (the Scherrer constant) is set to 0.89 (as in most polymer systems [52]), λ is the wavelength used, 2θ is the position of the (hkl) reflections, and β_hkl_ = (B_hkl_^2^ − b_o_^2^)^1/2^, with B_hkl_ being the peak width at half-maximum intensity and b_o_ being the instrumental resolution.

The small-angle X-ray scattering (SAXS) investigations were performed using an MBraun camera that utilized a conventional Kratky collimation system (HECUS-MBraun Graz X-Ray Systems, Graz, Austria). The front of the camera was directly mounted on top of the tube shield of a stabilized Philips PW 1830 X-ray generator. The X-ray tube was operated at a power of 1.5 kW. CuKα radiation was used. Scattered radiation was recorded in an acquisition time of 1200 s, using an MBraun linear position-sensitive detector, model PSD 50. The detector had 1024 channels, with a channel-to-channel distance of 52 µm. SAXS measurements performed in the direction parallel to the fiber axis were collected in the range of 0.02 ≤ s ≤ 0.8 (nm^−1^) (s = 2sinθ/λ, where 2θ is the scattering angle and λ is the X-ray wavelength). Analysis of the SAXS data was carried out using a normalized one-dimensional correlation function [53]:

(1) $$\gamma \left(r\right)=\frac{\int_{0}^{\infty }I\left(s\right)s^{2}\cos(2\pi rs)ds}{\int_{0}^{\infty }I\left(s\right)s^{2}ds},$$

where I(s) is the scattering intensity and “r” represents the distance in real space. Prior to integration, the data were extrapolated to zero and high angles, according to the procedure described in earlier works [54,55]. The structural parameters (long period (L), thicknesses of the crystalline (l_C_), and amorphous (l_A_) layers were obtained according to Strobl and Schneider [53].

## 3. Results and Discussion

### 3.1. Morphological Analysis

**Figure 1:**
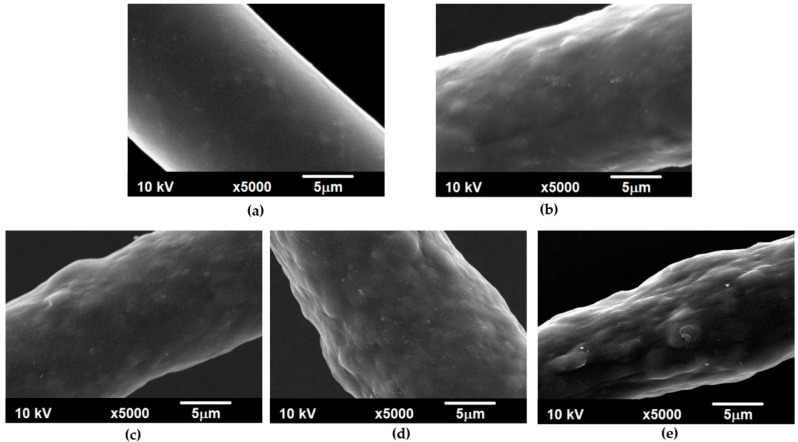
SEM images of the surfaces of the poly(butylene terephthalate) (PBT) fibers with different contents of reduced graphene oxide: (**a**) 0 wt %; (**b**) 0.5 wt %; (**c**) 1 wt %; (**d**) 1.5 wt %; (**e**) 2 wt % (V_take-up_ = 600 m/min).

Figure 1 shows how the surfaces of the fibers obtained under the same forming conditions changed with an increase in the amount of nanoadditive used.

The introduction of reduced graphene oxide into the polymer matrix caused a significant change in the morphology of the PBT fibers, which were characterized by a regular and smooth surface (Figure 1a). As the SEM images show (Figure 1b–e), the presence of rGO resulted in an appearance with various sizes of protrusions and unevenness on the surfaces of the fibers. However, even for a sample containing 2 wt % of rGO (Figure 1e), the resulting protrusions remained evenly distributed over the entire length of the fiber. The change in fiber morphology depending on the take-up velocity is shown in Figure S2. Regardless of the amount of the nanoadditive in the polymer matrix or the change in the fiber formation speed, the unevenness "hidden" under the skin layer of the polymer is visible on the surface. During microscopic observations, rGO was not observed to protrude beyond this layer in any of the obtained fibers. This "entrapment" of the nanoadditive increases the likelihood of being unable to remove it mechanically by detaching it from the surface of the fiber during further processing (for example, yarn formation).

**Figure 2:**
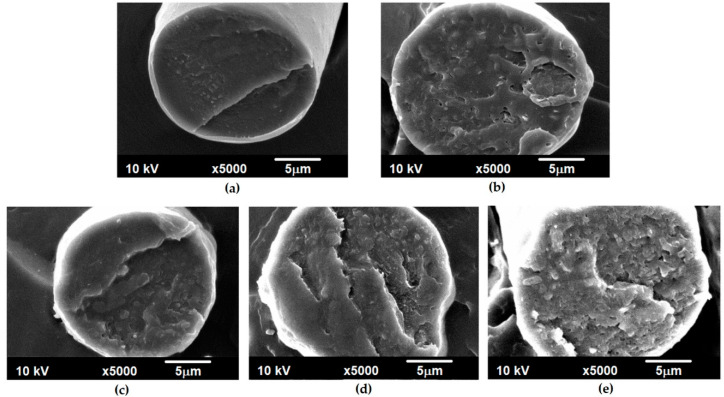
SEM images of the cross-sections of PBT fibers with different amounts of reduced graphene oxide: (**a**) 0 wt %; (**b**) 0.5 wt %; (**c**) 1 wt %; (**d**) 1.5 wt %; (**e**) 2 wt % (V_take-up_ = 600 m/min).

The microscopic observations of cross-sections of the obtained fibers were also performed (Figure 2).

For PBT fibers without the addition of reduced graphene oxide, virtually smooth cross-section surfaces were observed, with small cracks occurring in some places. With the introduction of the nanoadditive and an increase in its amount, the fracture surface cross-sections became more rough (Figure 2b–e). Nevertheless, regardless of the concentration of rGO in the polymer matrix, the nanoadditive did not form clearly visible agglomerates. Similar observations were made during the microscopic observations of the fibers formed at different take-up velocities, which contained 0.5 wt % of rGO (Figure S3).

The effect of rGO addition and forming conditions on the fiber diameter is presented in the Supplementary Materials (Figures S4 and S5).

### 3.2. X-ray Studies

**Figure 3:**
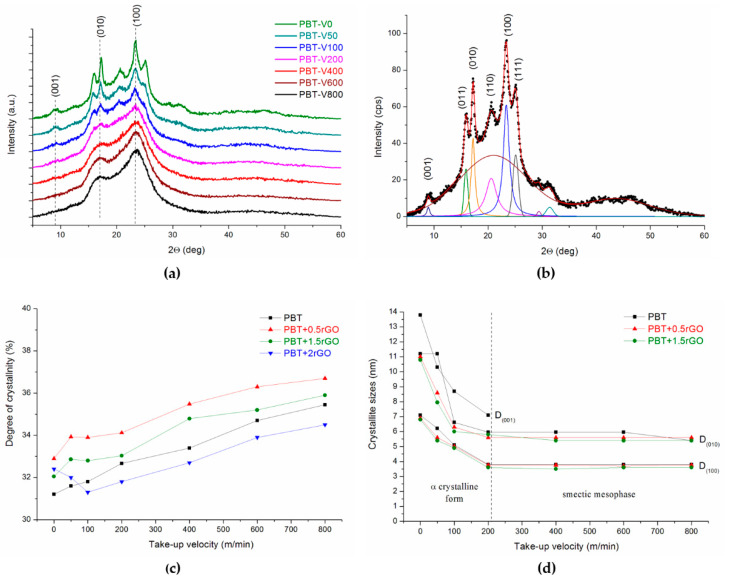
(**a**) Wide-angle X-ray scattering (WAXS) patterns of pure PBT fibers spun at different take-up velocities (the curves were shifted along the intensity axis for clarity); (**b**) deconvolution of the WAXS pattern of the PBT-V0 sample; (**c**) changes in crystallinity; (**d**) crystallite sizes of PBT fibers.

Figure 3a shows the series of WAXS patterns of the pure PBT fibers, taken at various take-up velocities, and those of PBT+1.5rGO fibers are shown in Figure S6. The WAXS patterns of the gravity spun fibers and fibers spun at a take-up velocity lower than 200 m/min exhibited six prominent diffraction peaks at 2θ Braggs angles of 9°, 15.9°, 17.2°, 20.6°, 23.4°, and 25.1°, corresponding to the diffraction planes of (001), (011), (010), (110), (100), and (111), which are characteristic of the α- crystalline form of PBT [56]. Most importantly, considering the structure of crystalline PBT, the peaks of planes (010) and (100) were parallel to the polymer chains oriented along the fiber axis, and the peak of plane (001) was perpendicular to the axis of the chain. The existence of these peaks unambiguously indicates the presence of α-crystals. On the diffractograms of the fibers spun at a take-up velocity above 200 m/min, only two broad interference peaks of planes (010) and (100) are visible. The absence of off-axis (mixed (hkl) like (110) or (111)) reflections indicates a translational disorder in the fiber direction of laterally aligned PBT macromolecules. In particular, the lack of reflex (001) shows that in the fibers spun at a high take-up velocity, no α crystalline phase was formed; instead, an ordered structure of a nematic or smectic type was created [57]. Ten years ago, Konishi and Miyamoto investigated the crystallization of PBT from a glassy state and revealed that PBT crystallizes through a mesomorphic phase, which they regarded as a smectic structure [58]. Later, the same authors, studying the crystallization of PBT from a melt, proposed a model for lamellar structure formation through the smectic phase [49]. The authors also observed the formation of the smectic phase in PBT films stretched at room temperature [45]. Recently, Tomisawa et al. [59] has observed the formation of a smectic phase by drawing PET fibers spun at 500–1500 m/min. However, to our knowledge, no one has yet observed the existence of a smectic phase in PBT fibers. Therefore, using other research methods, we next try to confirm the conclusions of the WAXS observations.

Quantitative development of the WAXS measurements was determined based on the deconvolution of the WAXS curves into scattering components from ordered and amorphous regions, which, following the procedure described in Section 2.5, facilitates the determination of the degree of crystallinity. An example deconvolution of the WAXS pattern for gravity spun pure PBT fibers is shown in Figure 3b, and that for the PBT-V600 fiber is shown in Figure S7. Figure 3c shows the dependence of the degree of crystallinity on the take-up velocity of fiber formation. For both pure PBT fibers and rGO-containing fibers, this parameter increases with an increase in the take-up velocity, but these changes are relatively small, within a range of 31–37% (Table S1). Some influence of rGO content on the degree of crystallinity of the investigated fibers can also be observed. Namely, for all spinning speeds, the degree of crystallinity of fibers containing a small amount of rGO (0.5 wt %) was greater than the crystallinity of fibers from pure PBT. As the rGO content increased, the crystallinity difference between the nanocomposite and pure PBT fibers decreased. For fibers containing 2 wt % rGO, the crystallinity was less than the crystallinity of the pure PBT fibers. 

The deconvolution of the WAXS curves allows us also to determine the half-width of the (001), (010), and (100) reflections, through which, according to Scherrer’s formula, the sizes of crystallites D_001_, D_010_, and D_100_ can be calculated, respectively (Table S2). Figure 3d summarizes the dependence of these parameters on the take-up velocity of fiber formation for pure PBT fibers and the two selected types of nanocomposite fibers. For the other types of nanocomposite fibers, the changes in crystallite sizes are very similar. To make this figure more readable, we present the changes in size D_001_ only for pure PBT fibers. As mentioned above, for take-up velocities above 200 m/min, due to the loss of order along the axis of the macromolecules, the peak from the plane (001) disappears, as does the size D_001_. Only the order of the polymer chains parallel to their axes is maintained in planes (010) and (100), which can be regarded as a smectic arrangement. The change in the ordering of macromolecules in the tested fibers is accompanied by a sudden decrease in the values of D_010_ and D_100_, which is clearly visible in Figure 3d.

**Figure 4:**
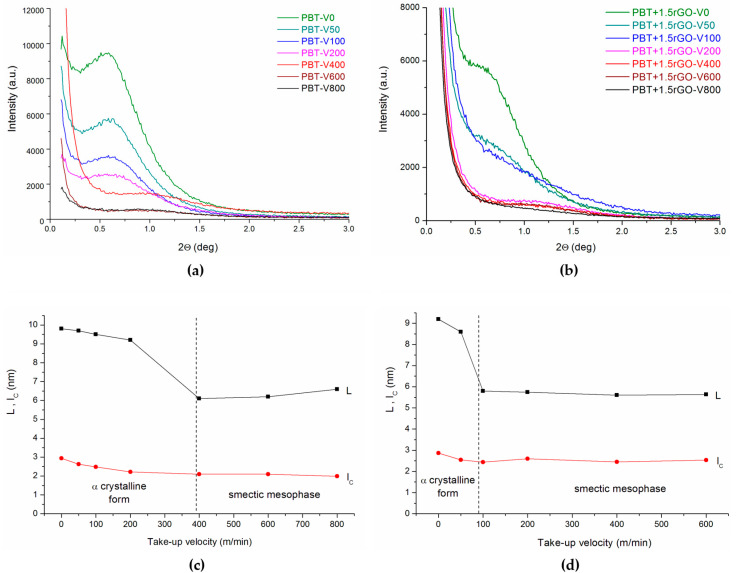
Small-angle X-ray scattering (SAXS) patterns of fibers spun at different take-up velocities: (**a**) PBT; (**b**) PBT+1.5rGO. Variations in the long period (L) and lamellar crystal thickness (l_C_) for fibers: (**c**) PBT; (**d**) PBT+1.5rGO.

It is well known that the α-crystalline phase of PBT forms a lamellar structure [49]. Hence, it seems reasonable to examine, using the SAXS method, the effect of fiber take-up velocity on the structure of fibers studied at the supermolecular level. Figure 4a shows a series of SAXS diffraction spectra for the pure PBT fibers. For fibers spun at a take-up velocity lower than 200 m/min, a distinct interference maximum at an angular position of 2θ ≈ 0.7° was observed. This maximum reflects the nearest-neighbour distance (i.e., the so-called long period) of the crystalline lamellar stacks. The SAXS patterns for fibers spun at a take-up velocity greater than 200 m/min are distinctly different from the others. The scattering intensity decreases, the interference maximum becomes very broad and poorly visible for fibers taken at the greatest take-up velocities, and its angular position moves towards much larger angles. A quantitative analysis of the SAXS measurements can be further obtained by applying the one-dimensional correlation function of the electron density fluctuations within the sample (Equation (1)), which yields the long spacing L, the average crystalline lamellar thickness l_C,_ and the amorphous layer thickness l_A_, where L = l_C_ + l_A_. The obtained values of these parameters are presented in Table S3. Figure 4c,d illustrate the changes in parameters L and l_C_ for fibers, for which the SAXS curves are presented in Figure 4a,b. For both types of fibers, the long period shows a remarkable decrease as the take-up velocity increases. All these findings suggest a profound transformation of the fiber structure at the supermolecular level. This transformation is undoubtedly associated with the formation of the smectic phase which was observed in the WAXS studies. The presence of rGO in the fibers caused a change in the supermolecular structure for fibers spun at 100 m/min.

### 3.3. DSC Studies

DSC calorimetric tests for fibers made of pure PBT and PBT fibers modified with reduced graphene oxide were carried out in a wide temperature range, from 0 °C to 260 °C.

**Figure 5:**
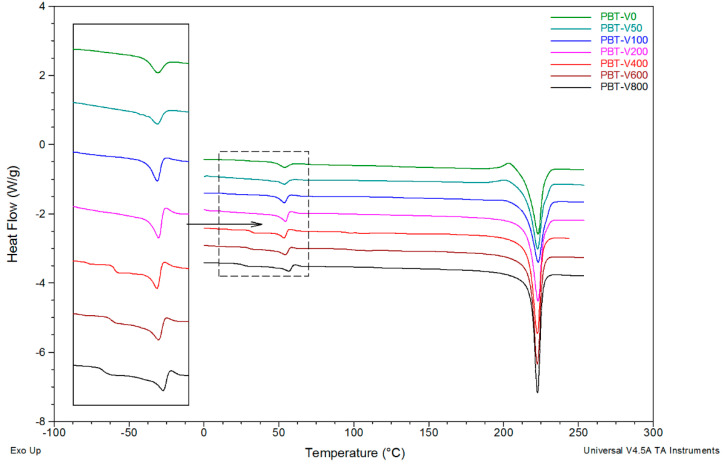
DSC curves of poly(butylene terephthalate) fibers formed at different take-up velocities.

**Figure 6:**
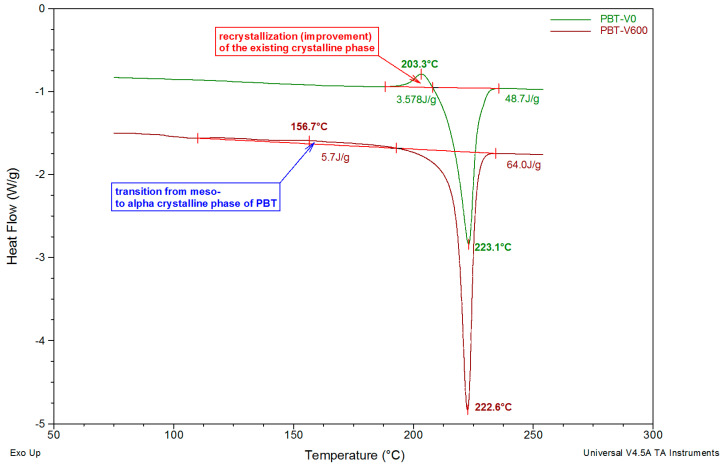
Illustration of the phase transitions that occur under the influence of heating: recrystallization within the existing PBT crystalline phase for fibers formed at take-up velocities from 0 to 50 m/min; phase transition of the smectic phase to the α-form for fibers formed at a take-up velocity above 400 m/min.

For pure PBT fibers, analysis of the DSC curves in the glass transition area revealed the appearance of two different glass transition temperatures for fibers formed at take-up velocities above 400 m/min (Figure 5). Based on the literature [45], we assumed that the additional glass transition temperature was related to the formation of an additional mesomorphic phase with smectic ordering. The T_g1_ signal of this phase appears on the DSC curve for PBT fibers formed at 400 m/min at a temperature of just over 31 °C (Table 1) and decreases monotonically to 25.5 °C at 800 m/min. In addition, for all studied PBT fibers, the T_g2_ signal of the amorphous phase was recorded at a temperature slightly above 50 °C. The T_g2_ value increases slightly as the take-up velocity increases, except for fibers spun at 200 m/min. The glass transition is associated with the appearance of the thermal effect of enthalpy relaxation (sometimes confused with melting due to endothermic peak [60,61]), which is characteristic for all polyesters. The appearance of this effect makes it difficult to precisely determine the T_g2_ values directly from the DSC thermograms. For PBT fibers formed at low take-up velocities, a characteristic exothermic peak appears at DSC curves, with a maximum at just over 200 °C, corresponding to the re-crystallization of the crystalline phase existing in the fibers, immediately preceding the melting transformation (Figure 6). It is the most pronounced (ΔH_r_ = 3.6 J/g) for gravity-spun fibers and completely disappears at a forming speed of 100 m/min. However, at 400 m/min and above, in the range of 120–190°, an extensive exothermic peak with a low intensity appears on the curves (enthalpy of 5 J/g). This is a thermal effect corresponding to the transition of the smectic phase present in the fibers into the most thermodynamically stable α-form of PBT. The discussed transition occurs when the fiber samples in the DSC cell are heated during measurement. The temperature corresponding to the peak maximum (T_r_) shifts clearly from 152.2 °C, at a forming speed of 400 m/min, to 173.6 °C for 800 m/min. Thus, as Figure 6 shows, the take-up velocity of fiber formation strongly affects the nature of the changes that take place in their structures under the influence of heating.

In the DSC thermograms, in the order of increasing temperature, a very strong endothermic melting peak of the fiber crystalline phase can also be seen. The minimum position of this peak (T_m_) changes very slightly (within 0.5 °C) for fibers formed at different take-up velocities, while its enthalpy (ΔH_m_) increases strictly monotonically from 48.7 J/g for gravity-spun fibers to 64.0 J/g for fibers formed at 800 m/min. This corresponds to an increase in the degree of crystallinity X_DSC_ as a function of take-up velocity. However, this increase is affected by the existence of the effect of recrystallization.

**Figure 7:**
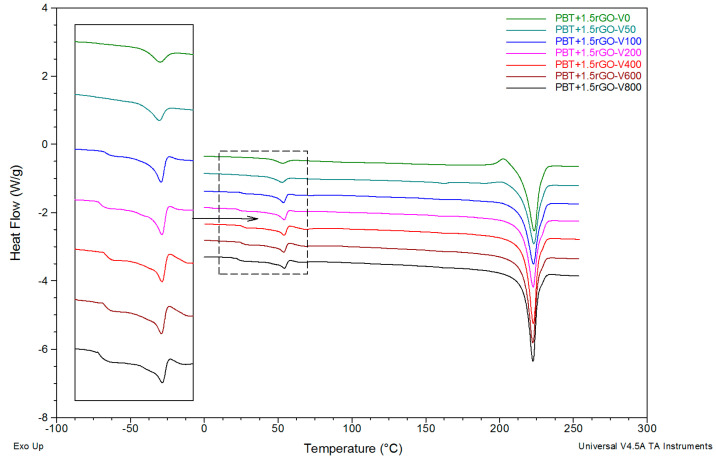
DSC curves of the PBT+1.5rGO fibers formed at different take-up velocities.

For PBT fibers modified with 1.5 wt % rGO, analysis of the recorded DSC curves (Figure 7, Table 2) leads to the same conclusions as above, with reference to the pure PBT fibers.

The main difference here is that the T_g1_ signal from the smectic phase already appears at 100 m/min, and its temperature position does not change by increasing the take-up velocity. The same regularity applies to the determined values of the glass transition temperatures of the amorphous phase T_g2_ and the minimum peak of enthalpy relaxation T_re_ (Table 2). It is worth noting that the temperatures T_g1_, T_g2,_ and T_re_, for fibers with the addition of rGO, are slightly lower than those for the analogous pure PBT fibers. With the appearance of the glass transition on the DSC curves originating from the smectic phase, there is also an extensive exothermic effect (peak), corresponding to its transformation into an α crystalline phase during heating. Notably, the enthalpy values of this effect are approximately 50% lower compared to those of the fibers without the addition of rGO. The degree of crystallinity for the modified fibers, calculated based on the value of melting enthalpy and taking into account the recrystallization phenomena, is slightly higher compared to that of the pure PBT fibers for most variants of the take-up velocity.

### 3.4. Effect of Spinning Conditions and rGO Content on Fiber Structure Development

To explain the observed changes in the supermolecular structure of the investigated fibers, it is necessary to analyze the process of polymer crystallization that occurs during the spinning of the fibers from the polymer melt. Polymer crystallization is a two-stage process involving the partial arrangement of polymer macromolecules initiated by nucleation and followed by subsequent crystalline growth.

The hierarchical structure of PBT fibers is formed as a result of their non-isothermal crystallization. In the fiber formation process, the molten polymer is extruded through the spinneret holes, after which the extruded stream is immediately drawn uniaxially. The polymer macromolecules are then subject to tensile and shear forces, under which they are straightened and oriented. Small bundles of oriented chains form rows of crystallization nuclei, whose orientation is consistent with the direction of fiber extraction. New chains attach to the surfaces of these nuclei, allowing the epitaxial growth of the lamellar crystallites. The crystal lamellae fill the space in the direction perpendicular to the row nuclei.

The crystallization process is highly dependent on the fiber formation parameters, PBT chain structure, and additives added to the polymer melt. The formation parameters have the greatest influence on the crystallization process, including the fiber take-up velocity, extruded melt temperature, and mass flow. These parameters determine the cooling speed of the solidifying stream and the orientation of the macromolecules, i.e., the two parameters that determine the process of crystallization. With a constant mass flow, an increase in take-up velocity leads to a reduction in fiber diameter (Figure S5). Faster-moving fibers of a lower diameter are cooled at a higher speed, which leads to a decrease in the crystallization rate. On the other hand, an increase in the take-up velocity, through an increase in orientation, results in a faster crystallization rate. Ultimately, the crystallization rate in the solidifying stream is the result of both these effects overlapping. At low take-up velocities, both effects offset each other [62]. Therefore, in the pure PBT fibers that we examined, at take-up velocities below 200 m/min, the α crystalline form was obtained. For fibers taken up at speeds higher than 200 m/min, the cooling speed of the crystallizing melt in the solidifying fiber stream increased. As a result, crystallization in these fibers occurred at a lower temperature, where the mobility of the chains decreased significantly. This led to a decrease in the number of macromolecules that were capable of attaching to the surface of the ordered nuclei and formed crystalline lamellae. At the same time, due to the longitudinal velocity gradient, the chains became oriented parallel to each other. As a result of crystallization under these conditions, the α crystalline form disappeared, and the content of the smectic type mesophase increased.

**Figure 8:**
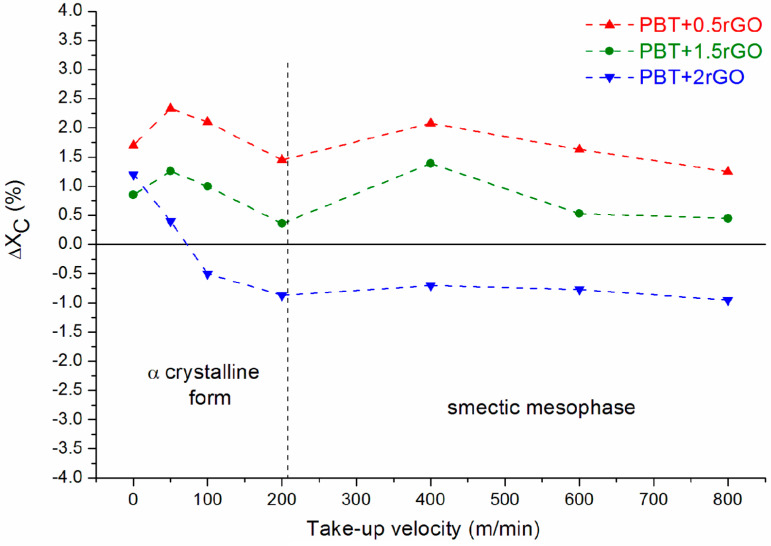
The dependence of the difference in the degree of crystallinity (ΔX_C_) of fibers containing rGO and pure PBT fibers on the take-up velocity.

The addition of rGO particles into the sheared polymer melts complicated the crystallization process and the morphology that subsequently formed. This can be attributed to the interactions between the polymer chains and the inorganic surfaces of the nanofiller, which leads to the formation of a significant volume fraction of “interfacial” regions with properties different from those of the bulk polymer, even at small loadings. It has been recognized that in the bulk far from the fillers, the local stress has the same value as in unfilled polymer [63]. Near the fillers, the stress is much higher, which can cause an increase in the orientation of some polymer chains during fiber spinning and can enhance the crystallization process. Thus, the polymer crystalizes differently when it is far from inorganic surfaces compared to when it is close to them. In some cases, confinement is introduced when the chains are restricted within the spaces between the nanoparticles [64]. Both types of nano-confinements affecting the supermolecular structure can be observed in the studied fibers, although their effects are rather subtle. To better observe the impact of rGO, effects associated with the spinning speed should be eliminated. This can be done by analyzing the difference in the degree of crystallinity of fibers containing rGO and pure PBT fibers. Figure 8 presents the dependence of the difference in crystallinity (denoted as ΔX_C_) on the take-up velocity for fibers with different levels of rGO content. This figure shows that for rGO content less than 2 wt %, rGO acts as a weak nucleating agent, slightly increasing the content of the ordered phase in the fibers. However, the nucleating properties of rGO play a role mainly in the crystallization of fibers spun at a very low velocity. In this case, even for fibers containing 2 wt % of rGO, the nucleating effect of this nanoadditive can be observed.

**Figure 9:**
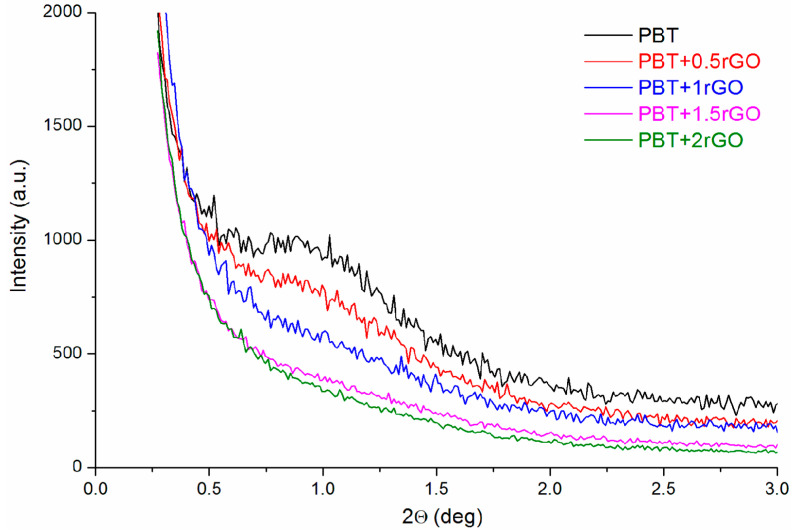
Comparison of the SAXS patterns of fibers with different amounts of rGO content spun at V = 800 m/min.

Nevertheless, it seems that rGO mainly hinders the crystallization of PBT by introducing steric obstacles that confine the ordering of the macromolecules of this polymer, especially for a high concentration of this nanofiller. When the rGO content increases, the space between the nanoparticles becomes smaller, and the polymer becomes restricted, with insufficient space for ordinary crystal formation. Hence, this effect is best observed in fibers containing 2 wt % rGO, spun at spinning speeds greater than 50 m/min, for which the degree of crystallinity is lower compared to the fibers from pure PBT. The influence of rGO on constraining the ordering of PBT chains can also be observed in the formation of the smectic phase that occurs in nanocomposite fibers at a lower take-up velocity than that for pure PBT fibers (Figure 4). This is not the only effect associated with the smectic phase. Figure 9 shows the SAXS curves of all fibers spun at the highest take-up velocity, 800 m/min. The maximum interference associated with the existence of the smectic phase clearly disappears for fibers containing 1.5 wt % and 2 wt % rGO. However, the existence of the peaks of planes (010) and (100) on the WAXS diffractograms of these fibers (Figure S6) clearly indicates that the PBT chains are ordered parallel to the direction of the fiber axis, albeit with no order along the fiber axis. This type of ordering is characteristic of the nematic rather than the smectic phase. This is a surprising result that was not observed for pure PBT fibers and resulted from the hindrance of PBT chain movement during the fiber formation process due to the presence of a significant amount of reduced graphene in the polymer melt.

### 3.5. Structural Evolution during Annealing

**Figure 10:**
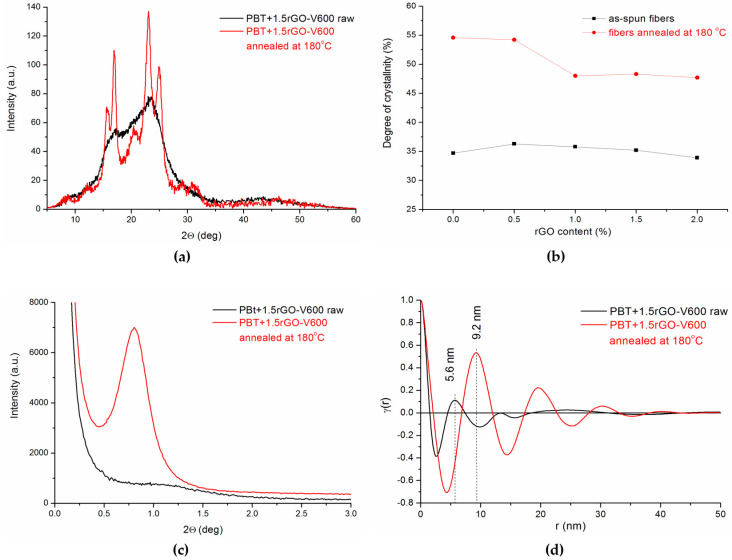
Comparison of (**a**) WAXS patterns; (**b**) crystallinity changes; (**c**) SAXS patterns; (**d**) correlation function for fibers PBT+1.5 wt %rGO-V600 both before (black line) and after annealing (red line) at 180 °C.

The smectic liquid crystalline phase has an intermediate structure between crystal and amorphous. For PBT, the smectic phase was observed in quenched PBT only by stretching it below room temperature [45]. It was also reported that the smectic structure transformed into the α-crystalline form of PBT via heating. Our research shows the possibility that the smectic phase forms as a result of the flow-induced crystallization that occurs during the spinning of fibers at high take-up velocities. To confirm these observations, we checked the behavior of this mesophase upon heating. All fibers that were spun at 600 m/min were selected for the experiment. These fibers were annealed at 180 °C for 1 h. Figure 10a shows a comparison of the WAXS diffraction patterns obtained before and after the annealing of the fibers containing 1.5 wt % of rGO. The curve of the annealed sample contains all the peaks that are characteristic of the α-crystalline form, which clearly demonstrates the expected transformation of the smectic phase into a crystalline form. The degree of crystallinity of the fibers after annealing is much greater than the degree of crystallinity of the as-spun fibers (Figure 10b) because, in the annealing process, the crystalline phase is formed from the pre-oriented polymer chains of the smectic phase. The sudden decrease in the degree of crystallinity in fibers containing more than 1 wt % rGO can be attributed to the previously discussed influence of rGO, thereby confining the process of polymer crystallization. The transformation of the smectic phase into the α-form is accompanied by the formation of a lamellar structure, which is manifested on the SAXS diffraction curves by the strong interference peak associated with this structure (Figure 10c). The correlation functions calculated for the annealed fibers (Figure 10d) have several maxima, which indicates the long-range regularity of the electron density changes, which is typical for lamellar structures.

**Figure 11:**
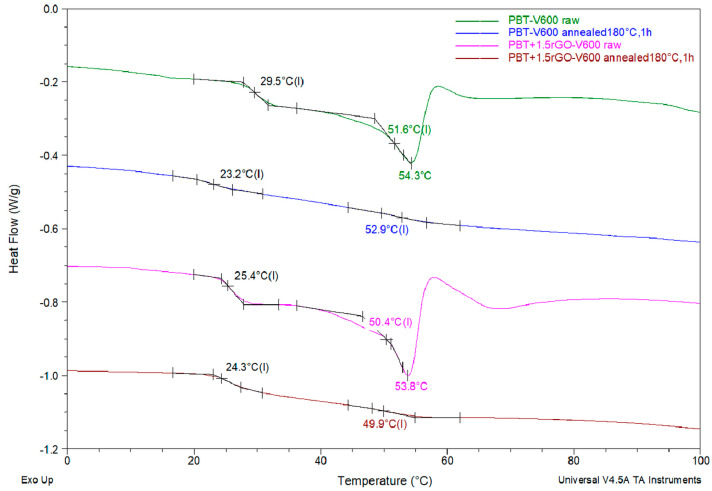
DSC curves of the studied fibers from PBT and PBT with the addition of 1.5 wt % of rGO both before and after annealing in hot air (180 °C for 1 h). Analysis of the glass transition temperature range. The inflection values of T_g1_ (black labels) and T_g2_ (colored labels) are marked on curves.

The transformation of the smectic phase into a stable α-PBT crystalline form was also confirmed by calorimetric studies. On the DSC curves recorded for the annealed fiber samples, an exothermic pre-melting effect corresponding to the transformation of the mesophase to the crystalline phase was no longer observed to such an extent (120–190 °C). Observations of the smectic phase transformation were also confirmed by an analysis of the DSC curves recorded in the region of the glass transition (Figure 11). Although the characteristic shift of the calorimetric signal line corresponding to the glassy transition of the smectic phase T_g1_ is observable on the fiber curves after heating, this line is clearly smaller and shifted towards lower temperatures compared to the same effect for the fibers before heating. However, the presence of a small peak of specific heat in the annealed fibers corresponding to the glass transition of the smectic phase suggests the presence of a small amount of untransformed mesophase in these fibers. In addition, as expected for the thermograms of annealed fibers, the enthalpy relaxation effect completely disappears. Consequently, the glass transition temperatures of the amorphous phase, T_g2_, can be easily determined. These values, after annealing the fibers at 180 °C, are slightly shifted, relative to the same values before fiber heating. For pure PBT fibers, the glass transition temperature, T_g2_, shifts slightly towards higher temperatures, while for fibers containing rGO, a reverse shift is observed. Due to the inability to precisely determine the glass transition temperature, T_g2_, for the as-spun fibers, changes in the values of this temperature for fibers after annealing cannot be considered. However, the observed changes in the glass transition temperature of the amorphous phase may themselves, in the future, be an interesting object of further research into the effects of the rGO modifier on the relaxation processes that occur in the structures of the fibers during heating.

## 4. Conclusions

Poly(butylene terephthalate) has many useful functional properties, analogous to PET. For this reason, PBT is now increasingly used as a typical common-use polymer. It is easier to process than PET and has very good fiber-forming properties. Therefore, it could be a potential substitute for PET fibers, which are currently the most important raw materials for the production of polymer fibers. The effects of spinning conditions and the presence of a small amount of reduced graphene oxide on the structural development of PBT fibers were analyzed by WAXS/SAXS, thermal (DSC), and microscopy techniques. Studies were performed for fibers containing four different concentrations of rGO, taken at different take-up velocities which ranged from 50 to 800 m/min. The obtained results showed that the take-up velocity of fiber formation has a major effect on the investigated fibers’ structures. The fibers spun at a small and medium take-up velocity contain the crystalline α-form, whereas the fibers spun at a high take-up velocity contain the smectic mesophase. For pure PBT fibers, the smectic phase appears for fibers formed at a take-up velocity above 200 m/min, whereas the rGO modified fibers already have a take-up velocity above 100 m/min. The addition of nanometric-sized rGO particles into the sheared polymer melts influences the crystallization process due to the attraction between the polymer chains and the inorganic surfaces of the nanofiller. However, studies have shown that the effect of this nanoadditive is subtle. Reduced graphene mainly hinders the crystallization of PBT by introducing steric obstacles that confine the ordering of the macromolecules of this polymer, especially for high concentrations of the nanofiller. Nevertheless, for rGO content levels of less than 2 wt %, it acts as a weak nucleating agent, slightly increasing the content of the ordered phase in fibers. For fibers containing 1.5 wt % and 2 wt % of rGO and spun at the highest take-up velocity (800 m/min), another interesting effect occurred. On the SAXS curves of these fibers, the disappearance of the interference peak characteristic of the smectic phase was observed. Since the WAXS diffraction patterns clearly indicate that the PBT chains are ordered parallel to the direction of the fiber axis, the disappearance of the SAXS peak suggests the transformation of the smectic phase into a nematic phase. This effect was not observed for the pure PBT fibers and was instead caused by the hindrance of PBT chain movement during the fiber formation process, resulting from the presence of a significant amount of reduced graphene in the polymer melt. 

These studies also show that, during annealing, the smectic phase transforms into the α-crystalline form. The degree of transformation depends on the rGO content.

## Figures and Tables

**Table 1:** Values of the characteristic temperatures of the glass transitions (T_g1_, T_g2_), enthalpy relaxation peak (T_re_), recrystallization (T_r_) and melting (T_m_), enthalpies of melting (ΔH_m_), recrystallization (ΔH_r_), and index of crystallinity (X_DSC_) of studied PBT fibers, determined based on DSC measurements.

Sample	T_g1_ (°C)	T_g2_ (°C)	T_re_ (°C)	T_r_ (°C)	ΔH_r_ (J/g)	T_m_ (°C)	ΔH_m_ (J/g)	X_DSC_ (%)
PBT-V0	–	50.7	54.0	203.3	3.6	223.1	48.7	31.1
PBT-V50	–	50.2	53.6	200.5	2.2	222.8	52.7	34.8
PBT-V100	–	50.2	53.6	–	–	223.1	54.6	37.7
PBT-V200	–	52.6	54.4	–	–	223.1	60.3	40.6
PBT-V400	31.2	51.4	53.5	152.2	5.2	222.6	61.1	38.6
PBT-V600	29.5	51.7	54.3	156.7	5.7	222.6	64.0	40.2
PBT-V800	25.5	53.5	56.7	173.6	4.8	222.6	61.8	39.3

**Table 2:** Values of the characteristic temperatures of glass transitions (T_g1_, T_g2_), the enthalpy relaxation peak (T_re_), recrystallization (T_r_) and melting (T_m_), enthalpies of melting (ΔH_m_) and recrystallization (ΔH_r_), and index of crystallinity (X_DSC_) of the studied PBT+1.5rGO fibers, determined based on the DSC measurements.

Sample	T_g1_ (°C)	T_g2_ (°C)	T_re_ (°C)	T_r_ (°C)	ΔH_r_ (J/g)	T_m_ (°C)	ΔH_m_ (J/g)	X_DSC_ (%)
PBT+1.5rGO-V0	–	50.0	53.1	202.5	4.5	223.4	52.4	33.5
PBT+1.5rGO-V50	–	49.5	52.9	200.6	1.0	223.3	51.2	35.1
PBT+1.5rGO-V100	25.6	50.6	53.7	–	–	223.0	56.5	39.6
PBT+1.5rGO-V200	22.8	51.5	54.1	170.9	1.4	222.8	56.9	38.9
PBT+1.5rGO-V400	25.4	51.5	53.3	166.6	2.4	222.1	59.5	40.0
PBT+1.5rGO-V600	25.1	50.7	53.8	171.0	2.1	222.5	63.1	42.7
PBT+1.5rGO-V800	22.2	51.1	54.2	173.2	3.4	222.6	60.6	40.0

## Supplementary Materials

The following are available online at https://www.mdpi.com/2073-4360/12/7/1456/s1, Figure S1: Characterisation of graphene oxide and reduced graphene oxide; Figure S2: SEM images of the surface of PBT +0.5rGO fibers formed at different take-up velocities; Figure S3: SEM images of the cross-sections of PBT +0.5rGO fibers formed at different take-up velocities; Figure S4: Diameter distribution of PBT +0.5rGO fibers with different contents of reduced graphene oxide; Figure S5: Diameter distribution of PBT +0.5rGO fibers formed at different take-up velocities; Figure S6: WAXS patterns of PBT +1.5rGO fibers spun at different take-up velocities; Figure S7: Deconvolution of the WAXS pattern of the PBT-V600 sample; Table S1: Degree of crystallinity obtained by means of the WAXS method; Table S2: Dimensions of crystallite D_hkl_ in the direction perpendicular to the lattice planes (hkl) determined by means of the WAXS method; Table S3: Results of the SAXS measurement containing a long period L, crystalline l_C,_ and amorphous l_A_ layer thicknesses.
