# Supplementary material for: Supermolecular Structure of Poly(Butylene Terephthalate) Fibers Formed with the Addition of Reduced Graphene Oxide
Source: Polymers (Basel). 2020 Jun 29;12(7):1456. doi: 10.3390/polym12071456 (PMC7407616; doi:10.3390/polym12071456)
Supplement: Supplementary file 1 [file polymers-12-01456-s001.pdf]

polymers

MDPI

Supplementary Materials

Supermolecular structure of poly(butylene terephthalate) fibers formed with addition of reduced graphene oxide

Czesław Ślusarczyk*, Marta Sieradzka, Janusz Fabia and Ryszard Fryczkowski

Institute of Textile Engineering and Polymer Materials, University of Bielsko-Biala, Willowa 2, 43-309 Bielsko-Biala, Poland; msieradzka@ath.bielsko.pl (M.S.); jfabia@ath.bielsko.pl (J.F.); rfyczkowski@ath.bielsko.pl (R.F.)

* Correspondence: cslusarczyk@ath.bielsko.pl (C.Ś.)

Received: date; Accepted: date; Published: date

Table of content:

S1. Characterization of reduced graphene oxide

S2. Morphological analysis

S3. X-ray studies

S1. Characterization of reduced graphene oxide

In Figure S1a are shown WAXS patterns of graphene oxide and reduced graphene oxide. Graphene oxide is characterized by a sharp peak at an angle of 2Θ=11.2°. Based on Bragg's equation, $n\lambda=2d\sin\theta$, where n is an integer, λ is the wavelength, d is the interlayer spacing, and θ is the scattering angle, the distance between the layers for GO was determined, and is 0.79 nm. Because of thermal reduction, the characteristic peak of graphene oxide disappears, while a broad and low intensity peak appears at an angle of 2 Θ=23.9°. This shift is associated with a reduction in the interlayer distance in reduced graphene oxide to 0.37 nm, which occurred as a result of the removal of oxygen-containing functional groups [1]. The reduction processes caused a decrease the number of layers, and the average number of layers in rGO is 6, compared to 23 in GO.

The characteristic bands for GO and rGO are visible in the FTIR spectrum (Figure S1b). The significant changes are observed in the bands at 3700-2200 cm^{-1} and in the fingerprint region (1500–400 cm^{-1}), where the bands characteristic for oscillators of less stable oxygen-functional groups are visible. Although the thermal reduction was carried out, the characteristic bands for carbonyl (at 1745 cm^{-1}) and carboxyl groups (at 1290 cm^{-1}), which are described as more stable oxygen-functional groups are still present. The band at 1622 cm^{-1} corresponding to the aromatic C=C ring stretching can still be found. These results show that low-temperature thermal reduction is an efficient way to remove less stable oxygen-containing functional groups from graphene oxide. The above results were confirmed by EDS analysis (Figure S1c). After thermal reduction, the amount of oxygen in rGO was reduced by half.

Graphite powder with a grain size <20 μm was submitted to oxidation and reduction reactions. To determine how these processes affected the size of the obtained rGO, the lateral size was measured using a scanning electron microscope. The average lateral size of rGO was 16.7 μm, and the histogram of the lateral size is ranging from 12.5 to 27.1 μm (Figure S1d).

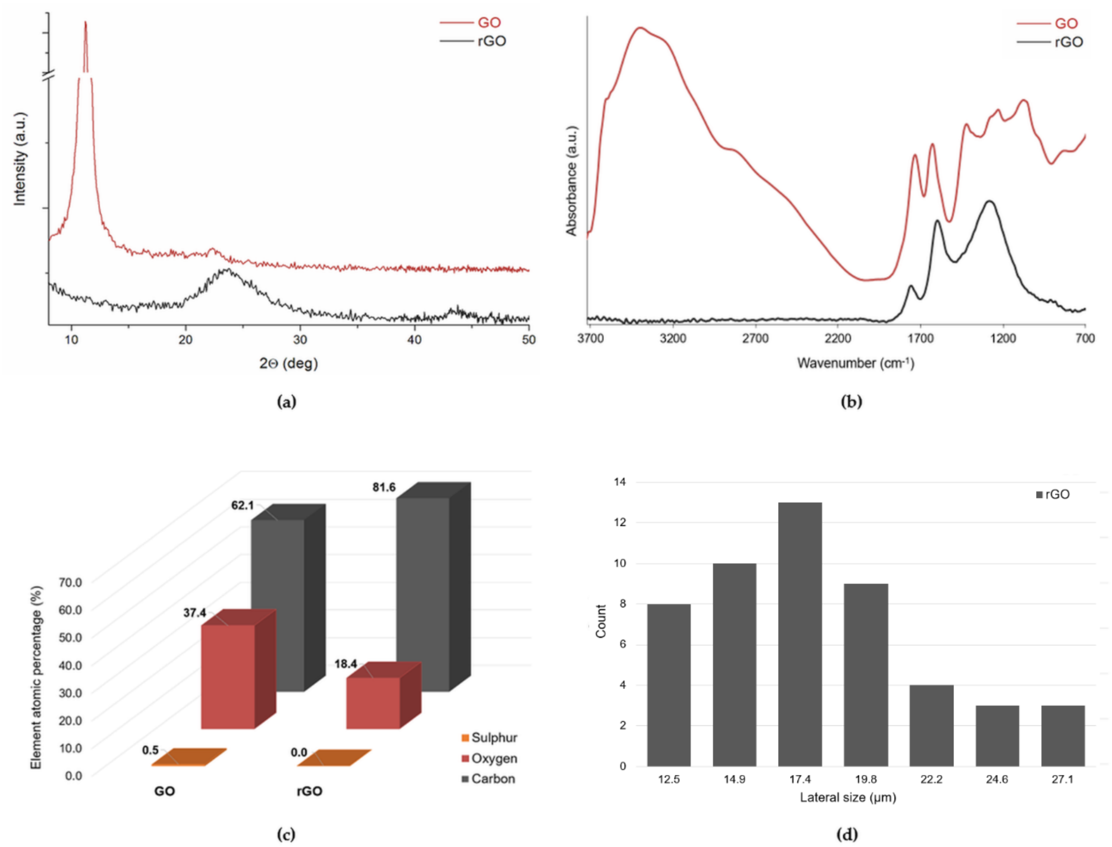

Figure S1. Characterisation of graphene oxide and reduced graphene oxide: (**a**) WAXS patterns; (**b**) FTIR spectra; (**c**) EDS analysis; (**d**) distribution of lateral size.

S2. Morphological analysis

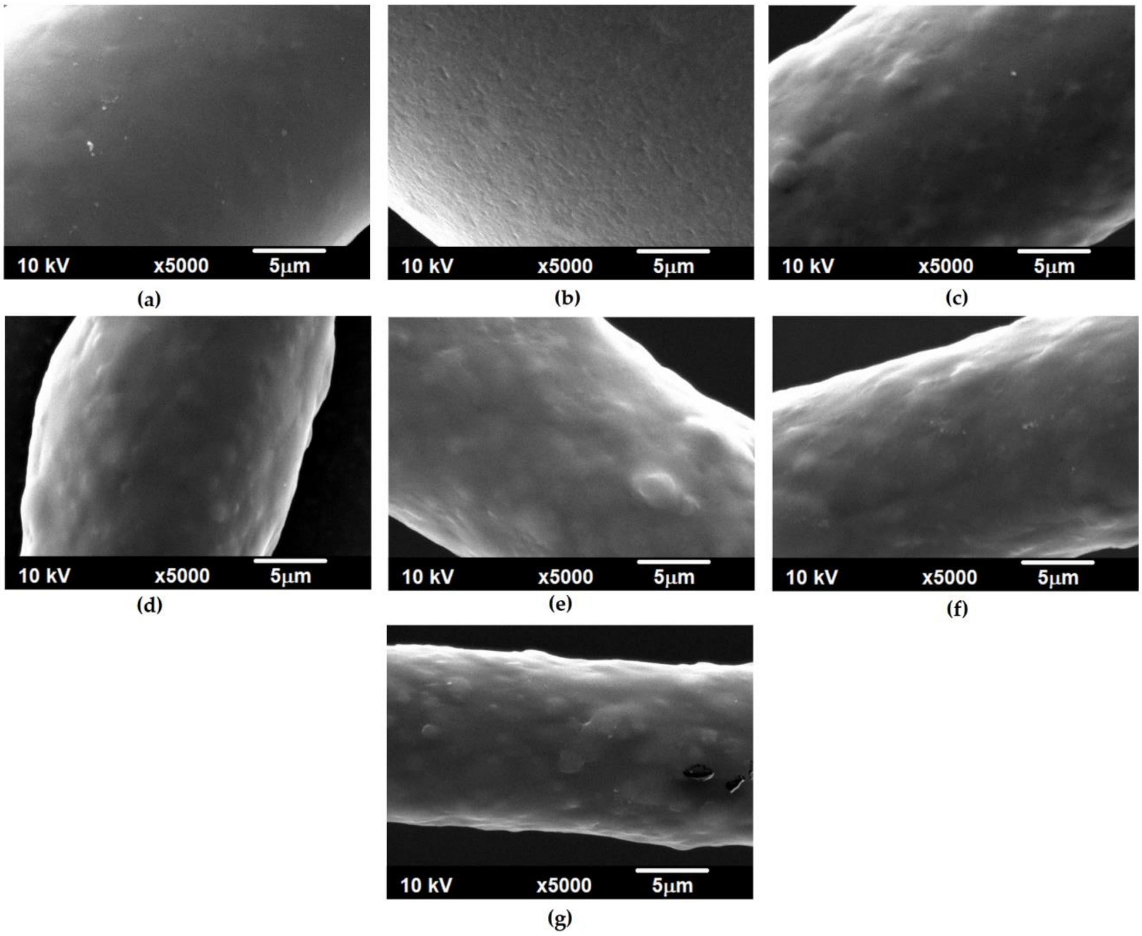

Figure S2. SEM images of the surface of PBT+0.5rGO fibers formed at different take-up velocities: (**a**) 0 m/min; (**b**) 50 m/min; (**c**) 100 m/min; (**d**) 200 m/min; (**e**) 400 m/min; (**f**) 600 m/min; (**g**) 800 m/min.

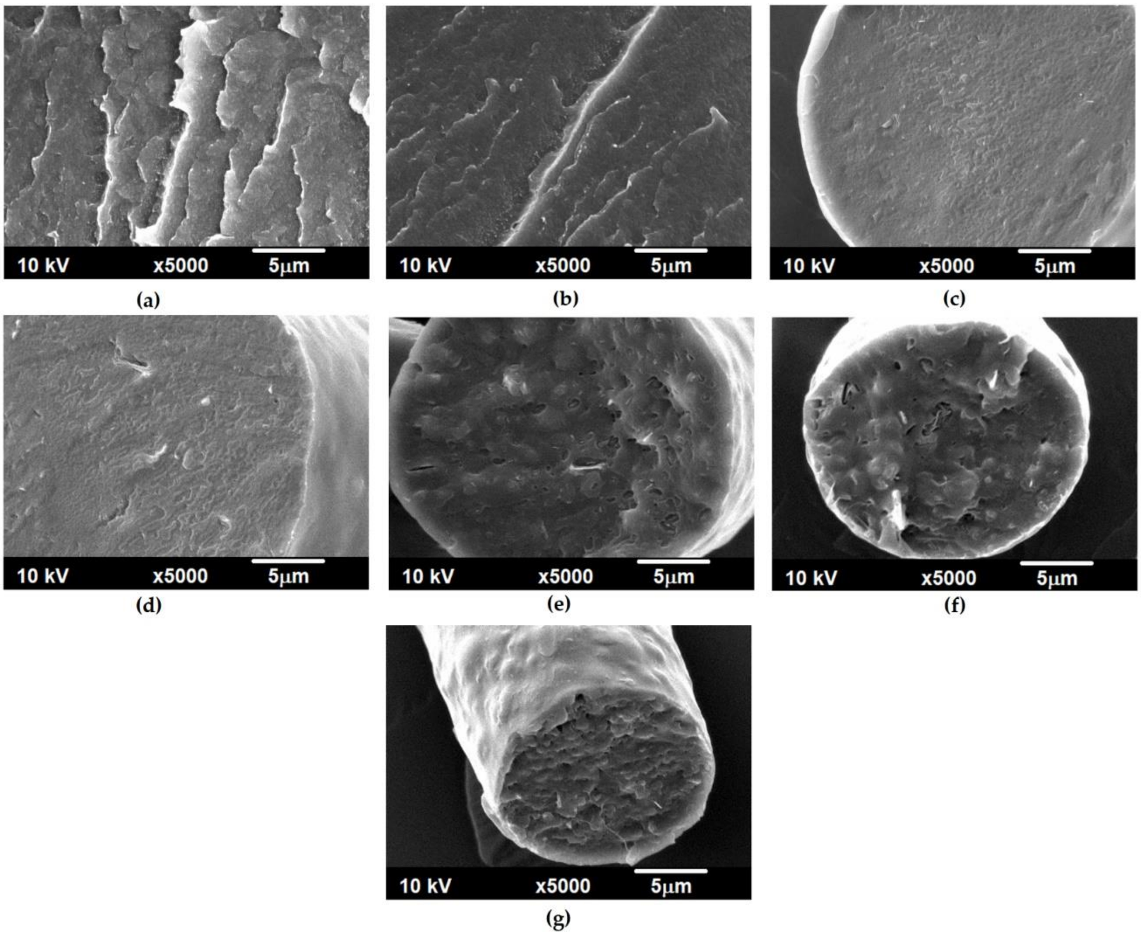

Figure S3. SEM images of the cross-sections of PBT+0.5rGO fibers formed at different take-up velocities: (**a**) 0 m/min; (**b**) 50 m/min; (**c**) 100 m/min; (**d**) 200 m/min; (**e**) 400 m/min; (**f**) 600 m/min; (**g**) 800 m/min.

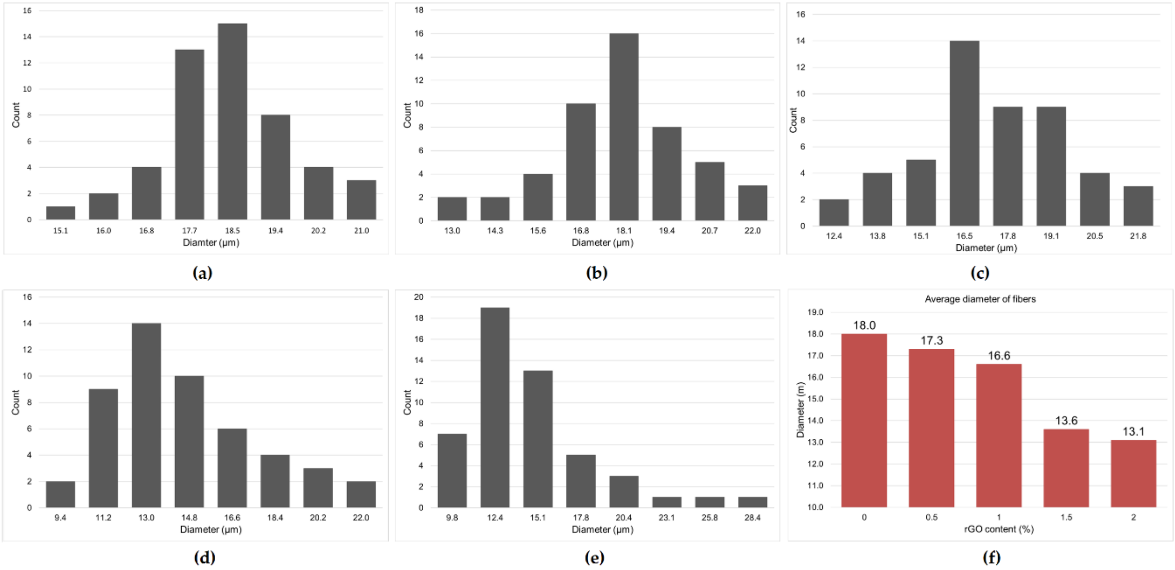

FigureS4. Diameter distribution of PBT+0.5rGO fibers with different contents of reduced graphene oxide: **(a)** 0 wt%; **(b)** 0.5 wt%; **(c)** 1 wt%; **(d)** 1.5 wt%; **(e)** 2 wt%; **(f)** average diameter of fibers.

The introduction of reduced graphene oxide into the polymer matrix caused a slight decrease in fiber diameter. It may be related to the fact that the surface of the fibers with the increase in the content of nanocomponents become more and more irregular and uneven. For this reason, the differences in diameter of fiber may occur.

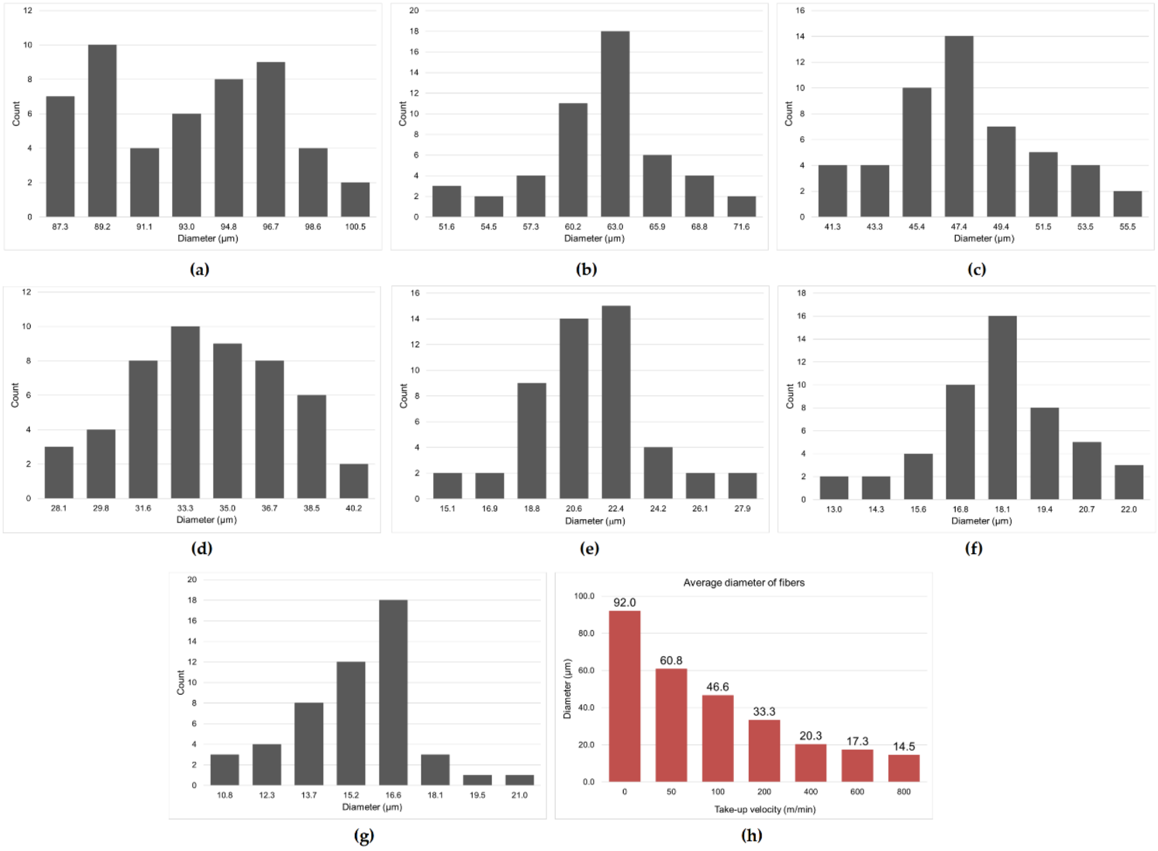

Figure S5. Diameter distribution of PBT+0.5rGO fibers formed at different take-up velocities: (**a**) 0 m/min; (**b**) 50 m/min; (**c**) 100 m/min; (**d**) 200 m/min; (**e**) 400 m/min; (**f**) 600 m/min; (**g**) 800 m/min (**h**) average diameter of fibers.

An increase in take-up velocity leads to a reduction in fibers diameter. The largest difference in diameter is observed between fibers formed with 0 and V = 50 m/min take-up velocity. For the rest of the fibers, a decrease in diameter is also observed, however, the higher the take-up velocity these differences are definitely smaller. Besides, it was observed that the diameter size distribution of PBT+0.5rGO-V0 fiber is bimodal, which is associated with the method of their formation (i.e. without stretching).

S3. X-ray studies

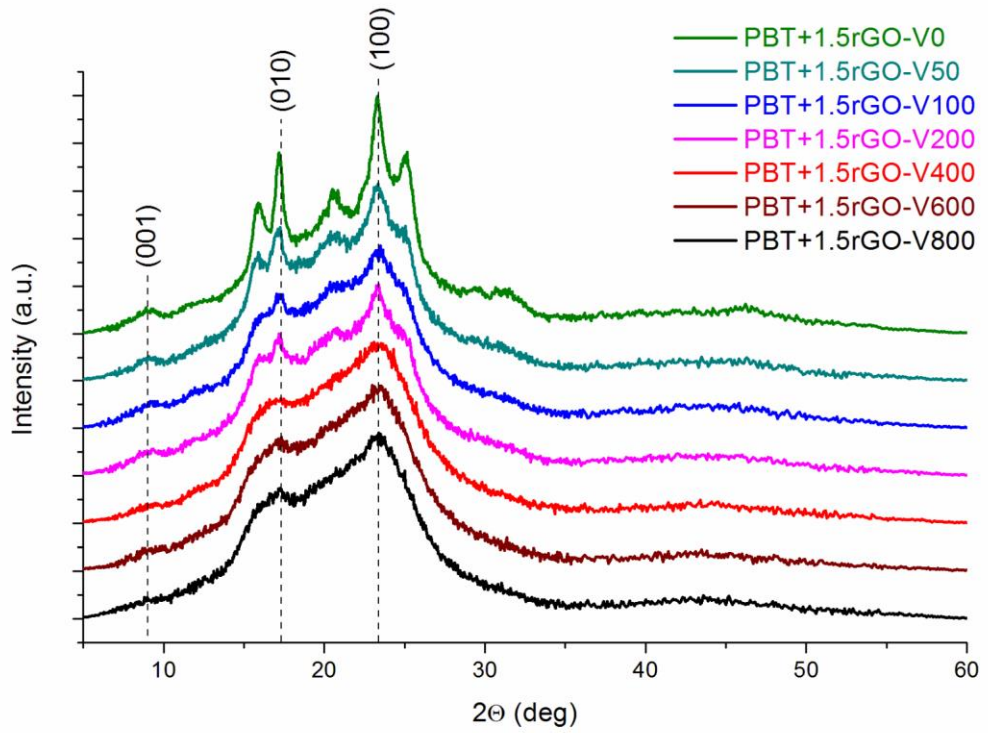

Figure S6. WAXS patterns of PBT+1.5rGO fibers spun at different take-up velocities (the curves were shifted along the intensity axis for clarity).

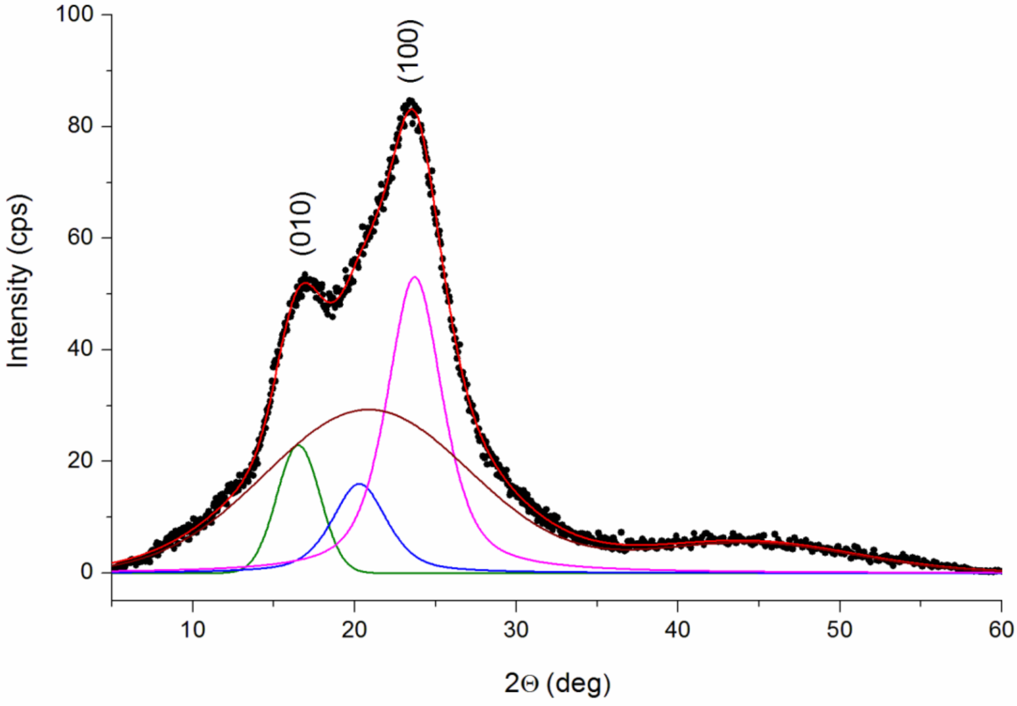

Figure S7. Deconvolution of the WAXS pattern of the PBT-V600 sample.

Table S1. Degree of crystallinity obtained by means of the WAXS method.

Take-up velocity, m/min	Degree of crystallinity, %				
	PBT	PBT+0.5rGO	PBT+1rGO	PBT+1.5rGO	PBT+2rGO
V-0	31.2	32.9	31.8	32.1	32.4
V-50	31.6	33.9	32.3	32.9	32.0
V-100	31.8	33.9	33.0	32.8	31.3
V-200	32.7	34.1	33.7	33.0	31.8
V-400	33.4	35.5	35.4	34.8	32.7
V-600	34.7	36.3	35.8	35.2	33.9
V-800	35.5	36.7	36.4	35.9	34.5

Table S2. Dimensions of crystallite D_{hkl} in direction perpendicular to the lattice planes (hkl) determined by means of the WAXS method.

Sample	D_{hkl}	Size of crystallites, nm						
		V-0	V-50	V-100	V-200	V-400	V-600	V-800
PBT	D_{001}	13.8	10.3	8.7	7.1	–	–	–
	D_{010}	11.2	11.2	6.6	6.0	6.0	6.0	5.4
	D_{100}	7.1	6.2	5.1	3.8	3.8	3.8	3.8
PBT+0.5rGO	D_{001}	14.7	11.4	9.7	8.3	7.2	–	–
	D_{010}	11.0	8.6	6.3	5.6	5.6	5.6	5.6
	D_{100}	6.9	5.6	5.0	3.8	3.8	3.7	3.8
PBT+1rGO	D_{001}	14.7	8.1	6.8	5.7	–	–	–
	D_{010}	11.2	9.1	5.8	4.7	4.7	4.8	4.7
	D_{100}	6.9	5.8	4.3	3.5	3.4	3.4	3.4
PBT+1.5rGO	D_{001}	11.4	8.8	6.6	6.0	–	–	–
	D_{010}	10.8	8.0	6.0	5.8	5.4	5.4	5.4
	D_{100}	6.8	5.4	4.9	3.6	3.5	3.6	3.6
PBT+2rGO	D_{001}	8.2	5.2	4.6	–	–	–	–
	D_{010}	11.4	6.3	5.4	5.4	4.70	4.7	4.7
	D_{100}	6.0	4.6	4.3	4.3	3.30	3.4	3.4

Table S3. Results of the SAXS measurement containing a long period L, crystalline l_C and amorphous l_A layer thicknesses.

**Sample**		**Parameters of lamellar structure, nm**						
		**V-0**	**V-50**	**V-100**	**V-200**	**V-400**	**V-600**	**V-800**
PBT	L	9.8	9.7	9.5	9.2	6.1	6.2	6.6
	l_C	2.9	2.6	2.5	2.2	2.1	2.1	2.0
	l_A	6.9	7.1	7.0	7.0	4.0	4.1	4.6
PBT+0.5rGO	L	9.2	8.8	5.8	5.2	5.2	5.0	5.1
	l_C	2.8	2.3	2.1	2.1	2.1	2.1	2.0
	l_A	6.4	6.5	3.7	3.1	3.1	2.9	3.1
PBT+1rGO	L	9.3	8.8	5.9	5.8	5.9	5.9	5.8
	l_C	2.7	2.4	2.2	1.9	1.9	2.0	2.2
	l_A	6.6	6.4	3.7	3.9	4.0	3.9	3.6
PBT+1.5rGO	L	9.2	8.6	5.8	5.7	5.6	5.6	–
	l_C	2.9	2.2	2.4	2.6	2.5	2.7	–
	l_A	6.3	6.4	3.4	3.1	3.1	2.9	–
PBT+2rGO	L	9.3	8.9	5.7	5.3	5.4	5.7	–
	l_C	2.6	2.5	2.4	2.2	2.0	1.9	–
	l_A	6.7	6.4	3.3	3.1	3.4	3.8	–

References

1. Sieradzka, M.; Ślusarczyk, C.; Fryczkowski, R.; Janicki, J. Insight into the effect of graphite grain sizes on the morphology, structure and electrical properties of reduced graphene oxide. *J. Mater. Res. Technol*. **2020**, *9*, 7059–7067. doi:10.1016/j.jmrt.2020.05.026.

© 2020 by the authors. Submitted for possible open access publication under the terms and conditions of the Creative Commons Attribution (CC BY) license (http://creativecommons.org/licenses/by/4.0/).
